# Autophagy in mycobacterial infections: molecular mechanisms, host-pathogen interactions, and therapeutic opportunities

**DOI:** 10.3389/fcimb.2025.1640647

**Published:** 2025-08-07

**Authors:** Jinyan Li, Haibo Feng, Dechun Chen, Huanrong Zhang, Yi Liao

**Affiliations:** ^1^ College of Animal Husbandry and Veterinary Medicine, Southwest Minzu University, Chengdu, China; ^2^ Institute of Qinghai-Tibetan Plateau, Southwest Minzu University, Chengdu, China

**Keywords:** mycobacterium, autophagy, molecular mechanism, host-pathogen interaction, host-directed therapy

## Abstract

Mycobacteria pose significant global health burdens, with *Mycobacterium tuberculosis complex* causing tuberculosis-a leading infectious killer claiming over 1.25 million lives annually-and NTM driving pulmonary and ulcerative infections, particularly in immunocompromised populations. Autophagy, a conserved cellular degradation pathway, serves as a critical mechanism of host defense against mycobacteria by delivering bacteria to the lysosome. As a response, mycobacteria have evolved intricate strategies to subvert or exploit autophagy for survival. Consequently, autophagy exhibits a dichotomous role in mycobacterial infection: functioning as a protective mechanism of host while simultaneously serving as a virulence determinant hijacked by bacteria for their survival. This review synthesizes current insights into the molecular mechanisms mediating host-initiated autophagy during mycobacterial infection, as well as the bacterial strategies for subverting or hijacking autophagic pathways. While autophagy may be hijacked by mycobacteria, substantial evidence from numerous studies demonstrates that autophagy-activating agents may be beneficial in restricting mycobacteria infection, even with multidrug-resistant strains. This review also systematizes promising agents that enhance autophagy to improve bacterial clearance. By synthesizing the latest research findings, this article aims to enhance our understanding of the intricate relationship between autophagy and mycobacteria, paving the way for efficient host-directed therapies (HDTs) against this severely harmful pathogen.

## 1 Introduction

Mycobacteria include the *Mycobacterium tuberculosis complex* and nontuberculous mycobacteria (NTM). The *Mycobacterium tuberculosis complex* can cause tuberculosis, which is a grave worldwide public health peril, claiming over 1.25 million lives each year. NTM can act as causal pathogens causing pulmonary and ulcerative human diseases (Crilly et al., 2020; Johansen et al., 2020; Kilinç et al., 2021). As successful intracellular bacteria, mycobacteria primarily resides within macrophages and phagocytes. Macrophages and phagocytes, which are the host cells of mycobacteria, activate multiple immune pathways to eliminate the invading bacteria. However, mycobacteria have developed diverse tactics to circumvent host immune elimination. The emergence of drug-resistant strains has reduced the cure rate of treating mycobacterial infections with antibiotics alone (Falkinham, 2018). Progress in the design of next generation antimycobacterial vaccines and agents demands a systematic understanding of the crosstalk between mycobacteria and host immune signaling pathways, which is complex and many aspects still need to be explored in more detail.

Autophagy represents an evolutionarily conserved biological process in eukaryotes, spanning from yeast to humans. Autophagy is employed to sustain cellular homeostasis by degrading organelles, proteins, nucleic acids, and lipids and recycling their components when the cell is subjected to nutrient deficiencies or invasion by pathogenic microorganisms (Lam et al., 2017). As a pivotal mechanism for sustaining cellular homeostasis, autophagy is involved in the prevention of multiple diseases. Besides, autophagy plays a vital role in the regulation of multiple immune responses, encompassing inflammation, innate and adaptive immunity, and antibacterial defenses. Therefore, it is not a wonder that under many pathological conditions perturbed autophagy can be implicated in a variety of diseases, including neurodegeneration, infection, inflammation, metabolic derangement, neoplasia and aging-related pathologies (Dikic and Elazar, 2018).

Within the context of host resistance to multiple intracellular bacterial pathogens, including mycobacteria, autophagy exerts a critical function by delivering endogenous and exogenous cargo to lysosome (Gutierrez et al., 2004; Yoshimori and Amano, 2009; Yuk et al., 2012; Manzanillo et al., 2013; Gomes and Dikic, 2014; Siqueira et al., 2018). Accumulating research show that autophagy affects both innate and adaptive immune responses (Yang et al., 2021; Nieto Ramirez et al., 2024). As a hedge, mycobacteria have also evolved various strategies to modulate autophagy and other immune pathways to evade host clearance (Chai et al., 2020). However, there is also study showing that autophagy is not relevant to the outcomes of mycobacterial infections. Autophagy genes protect the host against mycobacteria by reducing immune damage after infection rather than by enhancing autophagy (Kimmey et al., 2015). Although there is still debate about the specific role of autophagy in the eradication of mycobacteria, many autophagy-inducing drugs/agents can help better eliminate bacteria and reduce inflammatory damage, making autophagy induction a promising target for host-directed therapy (HDT) synergizing with existing therapies against mycobacteria (Yang, 2017; Kilinç et al., 2021; Zhao et al., 2023; Liu et al., 2024). In this review, we summarize the mechanistic insights into autophagy during mycobacterial infection and briefly discuss recent discoveries of autophagy-modulating agents that facilitate mycobacterial restriction.

## 2 Classification and processes of autophagy

Based on the different ways of transporting unwanted or harmful cytoplasmic cargo to lysosomes, autophagy in eukaryotic cells can be subdivided into three major types: microautophagy, molecular chaperone-mediated autophagy (CMA), and macroautophagy. During microautophagy, the lysosomal membrane invagination directly wraps intracytoplasmic cargo (Mijaljica et al., 2011) or these substances directly enter multivesicular bodies (MVBs) (Sahu et al., 2011). During CMA, intracytoplasmic unfolded proteins, recognized by molecular chaperones, enter the lysosome for degradation in a lysosomal-associated membrane protein 2A (LAMP2A)-dependent manner (Orenstein and Cuervo, 2010; Wang Y. et al., 2025). Macroautophagy is characterized by the appearance of a special double-membrane vesicle (DMV) called autophagosome that envelops the cargo to facilitate its delivery to the lysosome for degradation. Macroautophagy is the most extensively studied type and will be denoted as “autophagy” for brevity in the following part of this article. Autophagic degradation can be either non-selective or selective. Autophagic receptors serve as key determinants for selective autophagy, as they specifically recognize intracellular cargoes and mediate autophagosome formation. Cargoes include intracellular pathogens, mitochondria, endoplasmic reticula, peroxisomes, protein aggregates and lipid droplets, with their degradation via selective autophagy termed xenophagy, mitophagy, ER-phagy, pexophagy, aggrephagy and lipophagy, respectively.

The autophagy process is molecularly orchestrated by a series of proteins known as autophagy-associated proteins (ATGs). ATGs, first identified in yeast research, function through their mammalian homologs by forming five distinct complexes to mediate autophagy (Nakatogawa et al., 2009; Harnett et al., 2017).

ULK1 complex, which is composed of serine-threonine kinase, Unc-51 like kinase-1(ULK1), focal adhesion kinase family-interacting protein of 200 kDa (FIP200), ATG13 and ATG101 (Bento et al., 2015; Dikic and Elazar, 2018).

Class III phosphatidylinositol 3-kinase (PI3KC3) complex, which is composed of vacuolar protein sorting 34 (VPS34), autophagy and beclin 1 regulator 1 (AMBRA), ATG6, ATG14, phosphoinositide-3-kinase-regulatory subunit 4 (PIK3R4), and UV radiation resistance-associated gene protein (UVRAG) (Kametaka et al., 1998; Liang et al., 2006; Matsunaga et al., 2009; Bento et al., 2015; Dikic and Elazar, 2018).

WD repeat domain phosphoinositide-interacting (WIPI) proteins (Proikas-Cezanne et al., 2004; Polson et al., 2010; Harnett et al., 2017).

ATG12-ATG5-ATG16L complex (Mizushima et al., 1998, 1999; Bento et al., 2015; Dikic and Elazar, 2018).

Microtubule-associated 1 light chain 3 (LC3), a core autophagic protein, exists in two isoforms: LC3-I and LC3-II. LC3-II formed via conjugation of phosphatidylethanolamine (PE) to LC3-I by ATG3 and ATG7 (Kirisako et al., 1999; Weidberg et al., 2010; Bento et al., 2015; Dikic and Elazar, 2018; Frudd et al., 2018).

**Figure 1:**
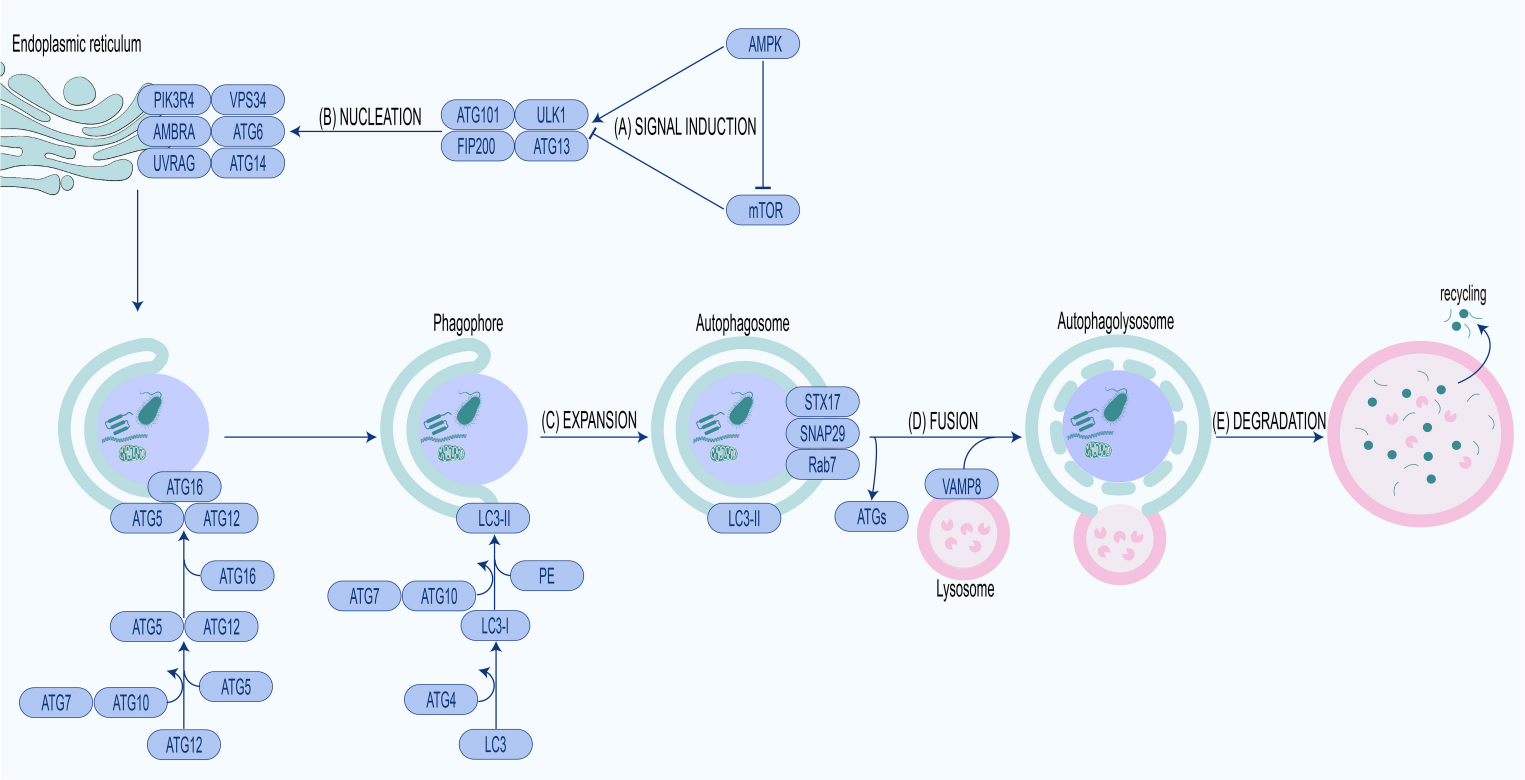
Molecular mechanism of autophagy.**(A)** Signal induction. Activation of AMPK or inhibition of mTOR leads to ULK1 complex activation and autophagy initiation. **(B)** Nucleation. Activated ULK1 complex phosphorylates PI3KC3 complex, producing PI3P, recruiting WIPIs and ATG12-ATG5-ATG16L complex. **(C)** Expansion. Recruited ATG12-ATG5-ATG16L complex conjugates PE to LC3-I to form LC3-II, expanding phagophore to form the autophagosome. **(D)** Fusion. Autophagosome fuses with lysosome to form autophagolysosome under the mediation of fusion proteins. **(E)** Degradation. The cargo in the autophagolysosome is degraded for recycling.

The above five complexes are sequentially involved in five stages of autophagy, including signal induction, nucleation, elongation, fusion and degradation (**Figure 1**).

### 2.1 Signal induction

The pivotal modulators of autophagy are two proteins with antagonistic effects, mechanistic/mammalian target of rapamycin (mTOR) and AMP activated protein kinase (AMPK) (Kim et al., 2011; Shang and Wang, 2011; Suzuki et al., 2025). mTOR is a serine/threonine protein kinase that regulates autophagy in response to hormones, nutrients, energy levels and oxygen content (Shang and Wang, 2011; Singh and Subbian, 2018). When nutrients are abundant, AMPK switches to an inactive state while mTOR undergoes activation. Activated mTOR binds to ULK1 and phosphorylates particular amino acid residues to inactivate it, thereby blocking ULK1-mediated autophagy initiation. Under nutrient deprivation, AMPK is activated, activating ULK1, ATG6, and VPS34 while inactivating mTOR, thereby enabling autophagy initiation (Hosokawa et al., 2009; Langer et al., 2024).

### 2.2 Nucleation

The activated ULK1 complex phosphorylates PI3KC3 complex and recruits it to phagophore assembly site (PAS). PAS is generally located in the endoplasmic reticulum, especially at the endoplasmic reticulum–mitochondria contact sites. The PI3KC3 complex recruited to the PAS catalyzes the production of phosphatidylinositol-3-phosphate (PI3P) (Nascimbeni et al., 2017). With the increase of PI3P in the PAS, WIPIs, as PI3P effector proteins, are also recruited to the PAS (Nair et al., 2010; Zhao et al., 2022). WIPIs can interact with ATG16L to facilitate recruitment of the ATG12-ATG5-ATG16L complex, which is indispensable for LC3 lipidation and autophagosome assembly (Strong et al., 2021).

### 2.3 Expansion

The ATG12-ATG5-ATG16L complex is an E3-like enzyme that plays a scaffolding role in LC3-I lipidation at sites where phagosomal membranes are to be formed (Fujita et al., 2008; Zhao et al., 2022). Upon autophagic induction, LC3-I is connected to PE through the catalysis of the E1-like enzyme ATG7 and the E2-like enzyme ATG3, forming LC3-II. Then LC3-II attaches to the inner and outer membranes of the phagosome and is removed from the autophagosome membrane by ATG4 prior to fusion with the late endosome/lysosome (Hussey et al., 2009; Carneiro and Travassos, 2013). Lipolysis of LC3 is in association with the ATG16L complex assembly as ATG12-ATG5 conjugation decreased significantly with the loss of LC3-II in ATG3-deficient cells (Sou et al., 2008; Lystad et al., 2019).

### 2.4 Fusion

Preceding the fusion of autophagosomes and lysosomes, ATGs are removed while fusion-associated molecules are recruited to autophagosomes. The autophagosomal membrane-anchored Syntaxin 17 (STX17), synaptosomal-associated protein 29 (SNAP29) and Rab7 interact with lysosomal membrane-anchored vesicle-associated membrane protein 8 (VAMP8), enabling fusion of autophagosomes with lysosomes and subsequent autophagolysosome formation (Furuta et al., 2010; Itakura et al., 2012; Hyttinen et al., 2013; Morelli et al., 2014; Zhao et al., 2021).

### 2.5 Degradation

When the autophagolysosome is formed, degradation of cargo inside autophagosome begins (Yang and Klionsky, 2010).

## 3 Autophagy against mycobacteria

### 3.1 Autophagy against *M. tuberculosis*

Host cells generate autophagy primarily by recognizing bacterial damage-associated molecular patterns (DAMPs) and pathogen-associated molecular patterns (PAMPs) (Deretic et al., 2015; Shibutani et al., 2015). When DAMPs and PAMPs are recognized, the cell generates signaling cascades that rapidly lead to the co-localization of autophagy machinery and cargo (van der Vaart et al., 2014a). The calcium signal is a DAMP. A recent study identified the tumor necrosis factor-like weak inducer of apoptosis (TWEAK) as pivotal mediator of calcium-associated autophagy. TWEAK binding to fibroblast growth factor-inducible 14 (Fn14) promotes calcium channel activation, leading to calcium influx and downstream activation of AMPK signaling to induce autophagy. Persistent TWEAK-Fn14 signaling also triggers mitochondrial ROS accumulation and cell death in late infection. Genetic depletion of Fn14 or TWEAK blockade suppresses autophagy and cell death, significantly enhancing mycobacterial survival in macrophages (Chen et al., 2022). The transcription of autophagic genes is further enhanced by the engagement of transcription factors, including nuclear factor-κB (NF-κB) and transcription factor EB (TFEB), which in turn promotes autophagy (van der Vaart et al., 2014b; Pastore et al., 2016). Notably, additional evidence from a separate study indicates that in non-immune epithelial cells, mycobacterial infection upregulates the expression of TLR2/4/7 to inhibit ROS, autophagy, and apoptosis in a MyD88-dependent manner, thereby promoting bacterial survival. This suggests that the TLR pathway exhibits dual regulatory roles in host defense across different cellular contexts (Singh et al., 2025).

In addition to DAMPs and PAMPs, cytokines also regulate host cell autophagy during *M. tuberculosis* infection. Stimulation of autophagic pathways by IFN-γ in mycobacteria infected macrophages causes colocalization of autophagy factors LC3 and mycobacterial autophagosome, indicating that intracellular mycobacteria may be target of host autophagy. IFN-γ also induces maturation of mycobacterial autophagosome, reducing intracellular viability of mycobacteria (Gutierrez et al., 2004; Songane et al., 2012; Dey et al., 2015; Watson et al., 2015; Ning et al., 2021; Yang et al., 2021). Subsequent study confirmed this finding and further revealed that although starvation and IFN-γ can trigger autophagy in host macrophage during *M. tuberculosis* infection, T helper (Th) 2 cytokines can reverse this anti-mycobacteria mechanism through AKT signaling and transcription 6 (STAT6) pathways respectively (Harris et al., 2007). It is seen that different cytokines may exhibit distinct autophagy-inducing effects. Furthermore, autophagy plays a role in additional anti-tuberculosis mechanisms such as increasing lysosomal bactericidal activity (Alonso et al., 2007; Purdy, 2011), modulating expression of scavenger receptors (SRs) (Bonilla et al., 2013) and increasing mycobacterial antigen presentation (Jagannath et al., 2009; Khan et al., 2021; Wu et al., 2022). In conclusion, these studies confirm the contribution of autophagy to host innate and adaptive immune defense against mycobacterial pathogens.

Furthermore, miRNAs may additionally modulate autophagy in mycobacteria-infected host cells. MiR-155 exhibits the opposite regulation of autophagy in different cells. In *M. tuberculosis*-infected dendritic cells, miR-155 impedes the formation of autophagosome and autophagolysosome (Etna et al., 2018), whereas in infected macrophages, miR-155 augments autophagic flux by interacting with Ras homologue enriched in brain (Rheb), which negatively regulates autophagy (Wang et al., 2013). The autophagy induced in *M. tuberculosis* infected macrophage is greatly repressed by miR-142-3p overexpression, which also prevents phagolysosome formation and enhances *M. tuberculosis* viability in macrophages. Additionally, by specifically targeting the 3’-UTR, miR-142-3p negatively regulates ATG16L1 and ATG4 expression, resulting substantial abatement of autophagy (Qu et al., 2021). Analogous to miR-142-3p, miR-874-3p and miR-129-3p also reduce macrophage autophagy through inhibiting ATG16L1 and ATG4 expression respectively (Qu et al., 2019; Luo et al., 2021). Apart from ATG16L1, ULK and ATG7 are also targets of miRNA to regulate autophagy in mycobacterial host cells. MiR-106a and miR-20a both downregulate ATG7 and ATG16L1 expression, whereas miR-106a also downregulates ULK expression to repress autophagy and facilitate *M. tuberculosis* survival (Guo et al., 2016; Liu et al., 2020). MiR-1958 exhibits similar inhibitory effect on autophagy and promotional effect on *M. tuberculosis* survival in macrophages. The mechanism is that miR-1958 directly targets the 3’UTR of ATG5, downregulates ATG5 expression, blocks autophagosome-lysosome fusion, impairs autophagic flux, and thus facilitates intracellular *M. tuberculosis* survival (Ding et al., 2019). ATGs are not the only target that miRNAs regulate to influence autophagy during mycobacteria infection. The flow of calcium ions from the endoplasmic reticulum toward the cytoplasmic matrix can cause autophagy. MiR-27a, which is increased in *M. tuberculosis* infection context, targets the ER-located Ca^2+^ transporter channel auxiliary subunit, downregulates Ca^2+^ signaling, thus inhibits autophagosome maturation, promotes *M. tuberculosis* survival (Liu et al., 2018). MiRNA possesses 3p and 5p arms. different arm of miR-30a plays contrary role on autophagy and anti-*M. tuberculosis* activity. MiR-30a-3p inhibits autophagosome and autophagolysosome formation and favors *M. tuberculosis* survival, whereas miR-30a-5p has the exact opposite effect (Behura et al., 2019, 2021). Similar to miR-30a, the role that the 3p and 5p arm of miR-125a plays in modulating is also opposite. MiR-125a-3p inhibits autophagy activation and anti-mycobacteria effect by targeting UVRAG, miR-125a-5p enhances autophagy activation and antimicrobial effects by inhibiting STAT3 (Kim et al., 2015; Wang Y. et al., 2020). MiR-17, another miRNA targeting STAT3, increases autophagy against *M. tuberculosis* by inhibiting upstream and downstream signaling of STAT3 (Kumar et al., 2016). Tumor necrosis factor (TNF)-like weak inducer of apoptosis (TWEAK), a factor that plays a role in promoting autophagy, is inhibited on expression by miR-889, resulting survival of *M. tuberculosis* (Chen et al., 2020). It has been reported that increased miR-23a-5p triggered by *M. tuberculosis* infection dramatically prevented autophagy in macrophages. Mechanistically, miR-23a-5p can inhibit TLR2/MyD88/NF-κB signaling by decreasing TLR2 expression (Gu et al., 2017). MiR-18a, belonging to the miR-17 family, demonstrates increased expression during *M. tuberculosis* infection and inhibits autophagy by downregulating ATM-AMPK signaling, resulting in increased bacterial survival (Yuan et al., 2020). A study has confirmed that miR-207 inhibited macrophage autophagy by directly binding to lysosome-associated membrane protein 2 (LAMP2), thus enhancing the survival of *M. tuberculosis* (Du et al., 2025). Damage-regulated autophagy modulator 2 (DRAM2), which interacts with UVRAG, is relevant to the promotion of autophagy. MiR144* can decrease DRAM2 expression and formation of autophagosomes, facilitate *M. tuberculosis* survival, by targeting 3’-untranslated region of DRAM2 (Kim et al., 2017). Additionally, a recent study revealed that miR-25-3p promotes macrophage autophagy by targeting dual specificity phosphatase 10 (DUSP10) to activate ERK1/2 phosphorylation, significantly increasing the expression of autophagy-related proteins like LC3-II and Beclin1, thereby reducing intracellular *M. bovis* survival (Yuan et al., 2023).

Interestingly, different mycobacteria differ in their ability to induce autophagy in host cells. proteomics revealed that *M. bovis* significantly increased the expression of 51 autophagy- and inflammation-related genes and activated the NF-κB pathway while *M. tuberculosis* only upregulated 8 energy metabolism-related proteins with weaker autophagy activation (Cai et al., 2023). Variations in the capacity to activate autophagy may not only partially dictate the survival outcomes of distinct mycobacterial strains but also underlie differences in their pathogenic potential.

### 3.2 Autophagy against NTM

As the mechanism of host resistance against pathogens, autophagy is also seen in nontuberculous mycobacterial infection (Daley and Winthrop, 2020). *M. avium* complex (MAC) is one of the most prevailing NTM species. Similar to *M. tuberculosis*-infected host cells, autophagy appears in MAC-infected macrophages through miR-125a-5p induced STAT3 (Wang Y. et al., 2020). Due to evolutionary conservation (Zhang et al., 2024), *M. marinum* can infect both humans and ectotherms and is often used as a model microbe of *M. tuberculosis* (Menon et al., 2025). Similar to *M. tuberculosis*, *M. marinum* also induces autophagy in host for the removal of intracellular bacteria (Chen et al., 2018; Kjellin et al., 2019). Researches on zebrafish models reveals that mediating by STING and p62, the DRAM1 functions downstream of TLR to activate autophagy, inducing defensive autophagy against *M. marinum* (van der Vaart et al., 2014a, 2014b; Zhang et al., 2020). Research on *M. marinum* infected dictyostelium discoideum reveals that, in addition to direct phagocytosis and removal of bacteria, the autophagy mechanism can repair disruption at the mycobacteria-containing vacuole in parallel with the endosomal sorting complex required for transport (ESCRT), thereby suppresses *M. marinum* proliferation (López-Jiménez et al., 2018). *M. smegmatis* and *M. fortuitum* are both fast-growing non-pathogenic NTMs, and both can induce strong autophagy independent mTOR pathway in host cells (Zullo and Lee, 2012). The autophagy induced by *M. smegmatis* in THP-1 macrophages relies on cell surface recognition receptor TLR2 but not bacterial ubiquitination, suggesting that host cells may remove *M. smegmatis* through non-selective autophagy (Bah et al., 2016). *M. terrae* is a slow-growing NTM that can cause intractable debilitating disease due to its antibiotic resistance (Wang et al., 2019). During *M. terrae* infection, autophagy induced by IL-17 is indispensable for antibacterial reaction in macrophages (Orosz et al., 2016).

### 3.3 Xenophagy against mycobacteria

In early studies, researchers believed that autophagy was a non-selective process, which indiscriminately wrapped all organelles and macromolecular complexes within a region of the cytoplasm into autophagosome for degradation. More recently, researchers observed that autophagy could act as a selective pathway that delivers specific organelles, invading organisms, or macromolecular complexes to autophagic machinery. During selective autophagy, recognition of cargo tends to be achieved by ubiquitylation that does not occur during non-selective autophagy (Gubas and Dikic, 2022). The ubiquitinated cargo is then bound to receptors proteins. Receptors contain LC3­interacting region (LIR) domains and ubiquitin­binding domains that anchor the LC3-containing phagophore to the cargo for engulfment (Kirkin and Rogov, 2019; Abdrakhmanov et al., 2020). Host selective autophagy for invading organisms is termed “xenophagy” (Galluzzi et al., 2017). Xenophagy is pivotal in orchestrating the host immune defense against mycobacteria because deletion of related genes allows the bacterium to proliferate abundantly (Shariq et al., 2023).

#### 3.2.1 Ubiquitination of mycobacteria

The mycobacterial ubiquitylation mediated by ubiquitin-ligating enzyme is the critical step for xenophagy origination. Two E3 ubiquitin ligases, parkin and smurf1, attach ubiquitin to bacteria during mycobacteria infection.

Parkin, as a member of the RBR ubiquitin ligase family, harbors multiple conserved domains, such as RING1, RING2, UBL, RING0, REP, and IBR (Dove and Klevit, 2017). Phosphorylation at S65 of the UBL domain and Ub elicits dramatic conformational changes and activation of parkin (Gladkova et al., 2018; Sauvé et al., 2018). In addition to its role in apoptosis, lipid metabolism, and inflammatory responses, parkin is involved in xenophagy against mycobacteria (Manzanillo et al., 2013; Romagnoli et al., 2023). *M. tuberculosis* utilizes its ESX-1 type VII secretion system to disrupt the phagosome membrane and enter the cytosol, where parkin catalyzes the K63-linked polyubiquitination to bacteria or bacteria-related membrane structures via its E3-Ub ligase activity. Autophagy receptors bind to ubiquitinated bacteria or membrane structures via the ubiquitin­binding domain and then recruit LC3-containing phagophore via the LIR domain. Parkin knockout in macrophage reduces LC3 lipidation and increases survival of *M. tuberculosis*, whereas parkin overexpression reduces bacterial proliferation (Manzanillo et al., 2013). Animal experiments have yielded consistent results. Parkin knockout mice exhibit more severe symptoms and higher mortality compared to wild-type mice. Meanwhile, parkin knockout increases mycobacterial replication and proliferation in the lung, spleen, and liver of mice (Manzanillo et al., 2013). These results suggest that ubiquitination and subsequent xenophagy facilitated by parkin play an essential role in the control of mycobacteria. Intriguingly, in macrophage infected with *M. tuberculosis*, only a portion of the intracellular bacteria is linked to the K63-linked Ub chains, the other portion is attached to the K48-linked Ub chains. These results suggest that other E3-Ub ligase are implicated in the ubiquitination of mycobacteria and relevant structures in addition to parkin.

Smurf1, an additional E3 ubiquitin ligase, mediates ubiquitination of intracellular mycobacteria. In addition to the E3-Ub ligase domain, smurf1 has a C2 phospholipid-binding domain, and both domains are involved in xenophagy against *M. tuberculosis*, as mutation in either of the two domains exhibits a deficiency in recruiting polyubiquitin, the autophagy receptor, the LC3 protein and the lysosome to *M. tuberculosis* relevant structures and exhibits increased mycobacterial survival in host cells (Franco et al., 2017). Analogous to parkin, smurf1 activity depends on ESX-1-driven translocation of *M. tuberculosis* from phagosomal compartments to the cytoplasmic matrix (Chandra and Philips, 2025). Contrary to parkin, smurf1 connects K48-linked Ub chains, not K65-linked ones, to *M. tuberculosis*. During chronic infection, smurf1-deficient mice display increased mycobacteria proliferation in the lungs and spleens compared to wild-types, whereas during acute infection, smurf1-deficient exhibit no effect on mycobacterial proliferation. Intriguingly, during acute infection, parkin-deficient mice display more *M. tuberculosis* proliferation. Whether these results are related to the different ubiquitin linkages catalyzed by parkin and smurf1 needs to be further explored.

Recently, tripartite motif 32 (TRIM32), another E3-Ub ligase belonging to the TRIM proteins family, was identified to be engaged in the ubiquitination of *M. tuberculosis* in host cells. TRIM32 knockdown in THP1 cells induces enhanced *M. tuberculosis* replication, owing to blocked bacterial ubiquitination, decreased autophagy recruitment and reduced autophagosome formation (Romagnoli et al., 2023). Another study identified a TRAF-like E3 ubiquitin ligase, TrafE, which integrates ESCRT and autophagy pathways by recruiting ALIX and Vps32 to damaged membranes during *M. marinum* infection. TrafE deficiency leads to reduced K63-polyubiquitination, impaired xenophagy, and premature host cell death, highlighting its critical role in membrane repair and bacterial restriction (Raykov et al., 2023). An *in vitro* autoubiquitination investigation revealed that the E3-Ub ligase, makorin ring finger protein 1 (MKRN1), in coordination with the ubiquitin-activating enzyme E1 (UBE1) and ubiquitin conjugating enzyme E2 D3 (UBE2D3), catalyzed the ubiquitination of *M. tuberculosis*, but not B. subtilis suggesting that MKRN1 may be a *M. tuberculosis*-specific E3-Ub ligase (Subrahmanian et al., 2020). However, whether MKRN1 catalyzes intracellular ubiquitination of *M. tuberculosis* or other mycobacteria needs to be further investigated.

In addition to being catalyzed by E3-Ub ligase, the *M. tuberculosis* can directly anchor to host ubiquitin chains through the mycobacterial surface protein Rv1468c, which harbors the eukaryotic-like ubiquitin-associated (UBA) domain (Chai et al., 2019). During Salmonella typhimurium infection, host galectin-8 identifies bacterially disrupted phagosomes, recruiting the autophagy receptor and LC3, inducing xenophagy (Thurston et al., 2012). These findings suggest that host can initiate xenophagy in a ubiquitin-independent manner. Considering that *M. tuberculosis* disrupted phagosomes may also be recognized by galectin (Schnettger et al., 2017; Morrison et al., 2023), it is not hard to understand that the host can also initiate xenophagy during mycobacteria infection through a galectin (rather than ubiquitin)-dependent manner (Bell et al., 2021). Furthermore, given the expanding number of eukaryotic-like effectors found in *M. tuberculosis* (Forrellad et al., 2013; Chai et al., 2018; Krause and Dikic, 2022), it is not implausible that *M. tuberculosis* may directly initiate host xenophagy by utilizing bacterial structural proteins that can be recognized by autophagy receptors or LC3 family members through protein–protein interaction.

#### 3.2.2 Autophagy receptors of xenophagy against mycobacteria

Beyond the pivotal roles of E3 ubiquitin ligases and diverse initiation mechanisms in xenophagy against mycobacteria, a set of specialized autophagy receptors further orchestrates the selective engulfment and elimination of intracellular mycobacteria, representing a critical downstream framework in this immune response. Autophagy receptors SQSTM1, CALCOCO2, NBR1, OPTN, and TAX1BP1 play crucial roles in mediating the attachment of intracellular mycobacteria to phagophores during xenophagy.

SQSTM1 was initially found to be a selective autophagy receptor for cytosolic protein aggregates (Komatsu et al., 2007; Pankiv et al., 2007; Tung et al., 2010; Chen et al., 2024). SQSTM1 contains not only a dimerization domain, a LIR domain, and a UBA domain, which are common to xenophagy receptors, but also a PB1 domain, a Ub ligase-interacting region and zinc finger. SQSTM1 self-oligomerization via the PB1 domain is indispensable for its function in selective autophagy (Itakura and Mizushima, 2011; Kim et al., 2016). To efficiently deliver cargo to the phagophore, SQSTM1 needs to interact with not only ubiquitin-modified cargo, but also other effector proteins (Clausen et al., 2010; Filimonenko et al., 2010; Chai et al., 2019; Zhang et al., 2019). The UBA domain of SQSTM1 is phosphorylated and ubiquitinated, which in turn binds to K63 linked cargo ubiquitin chains (Pilli et al., 2012; Lee et al., 2017; Lee and Weihl, 2017). The ubiquitination of SQSTM1 is catalyzed by effector proteins that bind to it. These effector proteins are primarily E3-Ub ligases like tripartite motif containing 50 (TRIM50), TNF receptor associated factor 6 (TRAF6), SMURF2, RNF166 and kelch-like ECH-associated protein 1 (KEAP1) (Komatsu et al., 2010; Lau et al., 2010; Heath et al., 2016; Lee et al., 2017). SQSTM1 participates in autophagic degradation of N-terminal arginylated proteins (Cha-Molstad et al., 2017; Zhang et al., 2018). SQSTM1 is involved in xenophagy of mycobacteria. SQSTM1 binds ubiquitinated Rv1468c protein on the surface of *M. tuberculosis* and brings *M. tuberculosis* to LC3-associated autophagosomes for xenophagy clearance (Chai et al., 2019). In *M. marinum* infected zebrafish model, SQSTM1 increases co-localization of LC3 with mycobacteria and inhibites bacteria multiplication (Zhang et al., 2019). Intriguingly, the antimycobacterial activity of SQSTM1 is not limited to xenophagy. SQSTM1 can deliver specific ribosomal and bulk ubiquitinated cytosolic proteins from the cytoplasm to autolysosomes for processing into molecules with antimycobacterial activity (Ponpuak et al., 2010). In addition, SQSTM1 is involved in the regulation of cyclic GMP-AMP synthase (cGAS)- Stimulator of Interferon Genes (STING) pathway. cGAS-STING pathway sensing to DNA induces phosphorylation of TBK1, which in turn activates IRF3, causing type-1 interferon expression. Phosphorylated TBK1 catalyzes the phosphorylation of SQSTM1, which induces the STING translocating to phagophores and degrading and avoids the overproduction of type-1 interferon (Prabakaran et al., 2018). TBK1 is a very specific molecule, which acts as a downstream of STING and participates in the generation of type-1 interferon (Wang et al., 2018) and also participates in mitophagy and xenophagy within mycobacteria-infected macrophages (Song et al., 2022). Given that type-I interferon, mitophagy, and xenophagy all modulate host-mediated clearance of mycobacteria, TBK1 emerges as a promising target for developing non-antibiotic therapeutics against these pathogens.

TAX1BP1 contains the LIR structural domain common to autophagy receptors for binding LC3, and unlike SQSTM1, TAX1BP1 does not contain the UBA domain. TAX1BP1 relies on the C-terminal overlapping Ub and myosin VI (MYO6) interacting domain to recognize ubiquitin (Ceregido et al., 2014; Whang et al., 2017; Fu et al., 2018). As with other autophagy receptors, TAX1BP1 targets ubiquitylated *M. tuberculosis* to LC3-containing phagophores, resulting clearance of bacteria (Budzik et al., 2020).

CALCOCO2 relies on the LIR and the unique CLIR to bind to LC3 and on the C2H2 zinc finger to recognize ubiquitin (Xie et al., 2015). CALCOCO2 delivers ubiquitinated *M. tuberculosis* to the phagosome, a process that requires the mycobacterial ESX-1type VII secretion system. BCG lacking ESX-1 cannot be delivered to the phagosome, and restoration of ESX-1 in BCG reverses this process (Watson et al., 2012).

OPTN, as an autophagy receptor, is involved in xenophagy, aggrephagy and mitophagy (Qiu et al., 2022). The LIR structural domain of OPTN is located between the two coil domains at the N-terminus, while the ubiquitin binding domains UBAN and zinc finger are located at the c-terminus. The UBAN domain preferentially recognizes linear ubiquitin chain (Li et al., 2018; Herhaus et al., 2019). The terminal coil domains of OPTN assemble into a heterotetrameric complex with the TBK1 C-terminus, thereby regulating OPTN function in selective autophagy (Morton et al., 2008; Li et al., 2016). OPTN has different phosphorylation sites and performs different functions. Phosphorylation of the S172 residue positioned close to the LIR by TBK1 leads to increased binding affinity of OPTN for LC3-family proteins. Phosphorylation of S473 in the UBAN domain by TBK1 potentiates OPTN binding to Ub, facilitating selective autophagy (Heo et al., 2015; Richter et al., 2016; Li et al., 2018). Phosphorylated OPTN exhibits co-localization with *M. tuberculosis* in macrophages (Budzik et al., 2020). Zebrafish model studies show that OPTN deficiency diminishes LC3-mycobacteria co-localization, thereby promoting bacterial proliferation, whereas OPTN overexpression augments LC3-bacterial co-localization, leading to reduced bacterial replication (Zhang et al., 2019). OPTN -deficient macrophages infected with high MOI mycobacteria exhibit enhanced cell death, reduced LC3-II levels, and altered Pro-IL-1β expression (Ramachandran et al., 2024). These results suggest that OPTN may play a role in xenophagy against mycobacteria.

NBR1 harbors a coiled-coil domain enabling its dimerization, a PB1 domain that interacts with the corresponding domain of SQSTM1, in addition to LIRs and a UBA domain (Lamark et al., 2003; Lange et al., 2005; Whitehouse et al., 2010; Rogov et al., 2014). Although NBR1 can act independently of and even antagonize SQSTM1 (Kirkin et al., 2009; Deosaran et al., 2013; Nishimura et al., 2024), the assembly of NBR1-SQSTM1 complex significantly enhances the efficacy of pexophagy and simaphagy (Deosaran et al., 2013; Migliano et al., 2024). Interaction with SQSTM1 may have altered the conformation of NBR1, thereby modulating its affinity for ubiquitin and LC3. Similar to SQSTM1 and TAX1BP1, NBR1 is engaged in xenophagy against *M. tuberculosis*, as NBR1 can recognize and bind the ubiquitinated *M. tuberculosis* surface protein PE_PGRS29 (Chai et al., 2019).

Exploring autophagy receptors plays a pivotal role in unraveling the molecular mechanisms of selective autophagy. With the increasing identification of these receptors, researchers have progressively turned their focus toward the interactions and regulatory networks among different autophagy receptors. Zebrafish models showed that the autophagic receptors OPTN and p62 function complementarily to independently restrict mycobacterial growth, while the autophagy modulator Dram1 can restore the association of autophagosomes with bacteria and lysosomal acidification even in the absence of both receptors, indicating that the three factors function independently yet synergistically in anti-mycobacterial immunity (Xie and Meijer, 2023). TBK1 phosphorylates autophagy receptors, enhancing their binding affinity to ubiquitinated substrates and promoting the occurrence of selective autophagy. Our study found that mitophagy competitively recruited TBK1 to mitochondria, reducing the translocation of TBK1 to mycobacteria and thereby inhibiting xenophagy (Song et al., 2022).

## 4 Mycobacteria regulate host autophagy for survival

**Figure 2:**
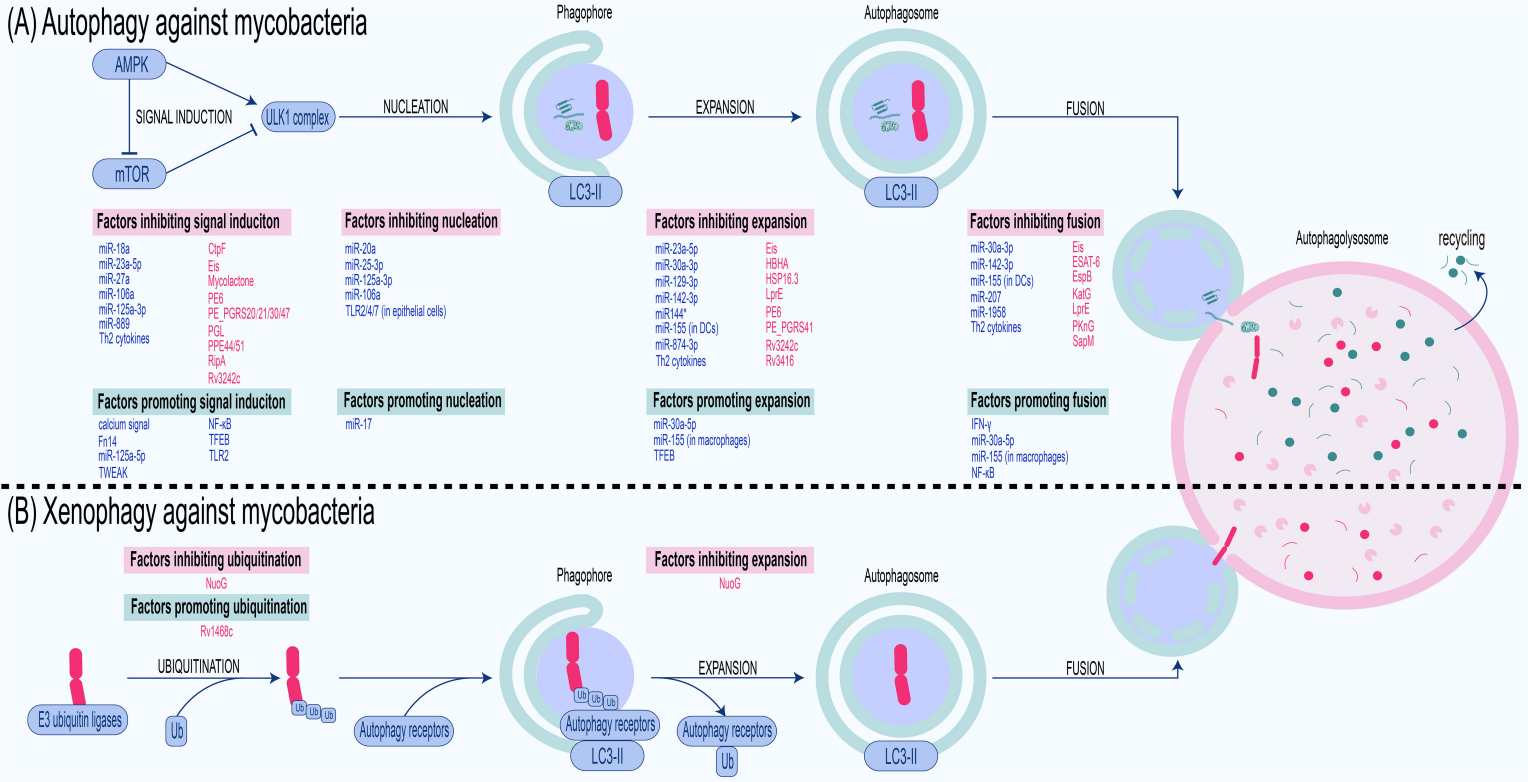
Antimycobacterial autophagy and xenophagy: processes and regulators. **(A)** Autophagy against mycobacteria. Cells sense nutritional or infection signals via the AMPK/mTOR pathway, activate the ULK1 complex, and recruit LC3-II to the phagophore with the assistance of ATGs. The phagophore embedded with LC3-II expands into an autophagosome, which engulfs components such as ​​bacteria, cytosolic organelles, and macromolecules​​. This autophagosome then fuses with the lysosome to form an autophagolysosome where the cargo is degraded. Thus, autophagy against mycobacteria also involves steps including signal induction, nucleation, expansion, and fusion, each of which is inhibited or promoted by factors derived from the host and bacteria. **(B)** Xenophagy against mycobacteria. Upon entering the host cytoplasm, mycobacteria are ubiquitinated by host ​​E3 ubiquitin ligases​​. The ubiquitinated bacteria are then recognized by ​​autophagy receptors​​. These receptors bind ubiquitinated pathogens via their UBA domain​​ while simultaneously anchoring to ​​LC3​​ on the phagophore through their LIR domain ​​. This dual binding recruits the pathogens to the phagophore, which subsequently elongates under the mediation of ​ATGs​​ to envelop the mycobacteria, forming an ​​autophagosome​​. The autophagosome then fuses with a lysosome to generate an ​​autolysosome​​, wherein the bacteria are degraded. Host-derived factors are marked in blue, whereas bacterial factors are marked in red.

While the host employs intricate autophagy mechanisms involving selective and non-selective autophagy to combat mycobacterial invasion, mycobacteria have evolved elaborate evasion strategies to subvert or exploit autophagy for their survival (**Figure 2**; **Table 1**), highlighting a dynamic interplay between host defense and pathogen evasion.

**Table 1:** Strategies of mycobacteria to regulate host autophagy.

Factors	Mechanism	Study model	References
CtpF	Activates mTOR	THP-1 and mouse peritoneal macrophages	(Garg et al., 2020)
Eis	Inhibits ROS production	BMDM	(Shin et al., 2010)
ESAT-6	Inhibits ROS production	J774 A.1	(Yabaji et al., 2020)
EspB	Inhibits IFN-γ-STAT1 axis	ANA-1	(Huang and Bao, 2016)
HBHA	Downregulates the expression of LC3 and Beclin-1, reducing autophagosome formation	A549	(Zheng et al., 2017)
HSP16.3	Inhibits autophagosome formation	RAW 264.7	(Yang et al., 2018)
KatG	Inhibits ROS production	RAW 264.7 and BMDM	(Siregar et al., 2022)
LprE	Reduces cathelicidin, ROS, IL-12 and IL-22, inhibiting phagolysosome fusion	THP-1, PBMC and BMDM	(Padhi et al., 2019)
mycolactone	Activates mTOR	THP-1 and BMDM	(Strong et al., 2022)
nuoG	Inhibits autophagosome formation and fusion with lysosome	THP-1	(Gengenbacher et al., 2016)
PE_PGRS20	Activates mTOR; Binds to Rab1A	RAW 264.7, THP-1 and BMDM	(Strong et al., 2020, 2021)
PE_PGRS21	Activates mTOR	RAW 264.7	(Strong et al., 2020)
PE_PGRS30	Activates mTOR	RAW 264.7	(Strong et al., 2020)
PE_PGRS41	Inhibits ATG-8 expression	THP-1	(Deng et al., 2017)
PE_PGRS47	Activates mTOR; Binds to Rab1A; Inhibits IL-1α-MAPK axis	RAW 264.7, THP-1 and BMDM	(Strong et al., 2020, 2021; He et al., 2025)
PE6	Induces phosphorylation of mTOR and ULK1	RAW264.7, THP-1	(Sharma N. et al., 2021)
PGL	Inhibits IRGM-mediated autophagy initiation	THP-1, U937	(Intemann et al., 2009)
PknG	Binds RAB14 and phosphorylates TBC1D4, inhibiting autophagosome maturation	U937 and BMDM	(Ge et al., 2022)
PPE44	Activates mTOR	RAW 264.7	(Strong et al., 2020)
PPE51	Activates mTOR	RAW 264.7	(Strong et al., 2020)
RipA	Activates PI3K-AKT-mTORC1 axis	RAW264.7	(Shariq et al., 2021)
Rv3242c	Inhibits ROS production	RAW 264.7, THP-1 and mouse peritoneal macrophages	(Mohanty et al., 2015)
Rv3416	Augments SUMOylation of Beclin-1 and LC3, inhibiting autophagosome formation	BMDC	(Anang et al., 2023)
SapM	Binds Rab7, blocking autophagosome-lysosome fusion	Raw264.7	(Hu et al., 2015)

Determinants that inhibit host autophagic clearance are often virulent factors of mycobacteria. As a classical virulence factor in mycobacteria, the ESX-1 secretory system is involved in the modulation of host autophagy. ESX-1 secretion-associated protein B (EspB) of *M. tuberculosis* reduces mRNA and protein levels of IFN-γ receptor 1 and decreases IFN-induced STAT1 phosphorylation in murine ANA-1 macrophage cells, thereby inhibiting LC3 expression and autophagosome maturation (Huang and Bao, 2016). Macrophages infected with ESAT-6-expressing mycobacteria exhibited elevated levels of SOD-2, decreased autophagosome-lysosome fusion, and increased survival of bacteria. These effects of ESAT-6 may be related to the SOD-2-mediated reduction of ROS (Yabaji et al., 2020). *M. ulcerans* is the etiological agent of Buruli ulcer, the third most prevalent mycobacterial infection worldwide. A study shows that *M. ulcerans* secretes mycolactone to induce necrotic cell death in macrophages, promoting bacterial escape while maintaining mTOR activation to suppress autophagy, as demonstrated by restored pathogenicity in a tetracycline-inducible mycolactone expression system (Strong et al., 2022). Phenolic glycolipid (PGL) is one of the virulence factors of *M. africanum*. Because Euro-American clades of *M. tuberculosis* lack the gene required for PGL biosynthesis, these clades are readily cleared by host autophagy enhanced by IRGM mutations. In contrast, this enhanced autophagic response is suppressed by *M. africanum*, which harbors an intact pks1/15 gene to produce PGL (Intemann et al., 2009). Since PGL reduces the synthesis of cytokines capable of inducing autophagy, like IL-6 and IL-12 (Reed et al., 2004; Cambier et al., 2017), it is hypothesized that the inhibition of autophagy by PGL may be partially mediated by the reduction of these cytokines.

The PE/PPE family, which can disrupt the host immune response, has a higher virulence compared to other mycobacterial proteins (Sharma et al., 2022). PE6 induces phosphorylation of mTOR and ULK1, thereby inhibiting the conversion of LC3I to LC3II and reducing autophagy (Sharma N. et al., 2021). Six PE/PPE family proteins with activating effect on mTOR and inhibitory effects on autophagy were screened by loss-of-function screening of an *M. tuberculosis* transposon mutant library. Expression of these proteins in *M. smegmatis* confirmed their autophagy inhibitory effect. The inhibition of autophagy conferred higher survival and replication of mycobacteria in host cells. The expression of these PE/PPE proteins varied under different stress conditions, suggesting that PE/PPE may confer adaptability of *M. tuberculosis* to a wide range of conditions (Strong et al., 2020). PE_PGRS20 and PE_PGRS47 inhibit autophagy initiation by binding to Rab1A of host cells, thereby increasing bacterial survival. Intriguingly, PE_PGRS20 and PE_PGRS47 also inhibit antigen presentation of host cells. This may be due to the fact that antigen presentation is dependent on phagocytic degradation of the mycobacteria by the host cell, and the inhibition of autophagy by PE_PGRS20 and PE_PGRS47 reduces the amount of antigen available for presentation from bacteria degradation (Saini et al., 2016; Strong et al., 2021). Additionally, PE_PGRS47 can synergistically inhibit macrophage autophagy and apoptosis by downregulating IL-1α secretion and inhibiting the MAPK signaling pathway (particularly p38 and ERK1/2), thereby promoting the survival of *M. smegmatis* within macrophages. This indicates that the multifunctional virulence factor PE_PGRS47 can evade host immunity such as autophagy through multiple pathways (He et al., 2025). PE_PGRS41 is also an autophagy inhibitor because PE_PGRS41 knock-in *M. smegmatis* reduced autophagy in infected macrophages (Deng et al., 2017). Under the pressure of adapting to the host, some PE/PPE family genes of *M. bovis* may be deleted or mutated, so that *M. bovis* has evolved a strategy to evade host autophagy without relying on PE/PPE family proteins. *M. bovis* inhibits autophagic clearance and promotes its intracellular survival by disrupting autophagosome-lysosome fusion and exploiting host energy metabolism remodeling (Cai et al., 2023).

In addition to the PE/PPE family, certain enzymes in mycobacteria can also inhibit autophagy, thereby facilitating bacterial survival. *M. tuberculosis*-secreted acid phosphatase SapM can inhibit autophagy by reducing phagosome-lysosome fusion and lysosomal acidification. SapM interacts with GTPase Rab7 through CT domain, thereby inhibiting autophagosome maturation and enhancing autophagosomes accumulation (Hu et al., 2015). RipA is a peptidoglycan hydrolase of *M. tuberculosis*. Survival of RipA-expressing *M. smegmatis* is increased in macrophages. Mechanistically, RipA interacts with the LIR of autophagy receptors and inhibits ULK by activating PI3K-AKT-mTORC1 signaling, thereby inhibiting antibacterial autophagy (Shariq et al., 2021). The enhanced intracellular survival (Eis) gene, which is an acetyltransferase, can increase the bacterial viability of mycobacteria, as literally indicated. Eis knockout *M. tuberculosis* causes infected macrophages to produce more ROS and autophagosomes, suggesting that Eis may inhibit autophagy by reducing ROS production. Genetic recombination assays showed that the inhibitory effect of Eis on ROS and autophagosomes production is dependent on its N-acetyltransferase domain (Shin et al., 2010). A calcium transporting P2A ATPase of *M. tuberculosis*, CtpF, inhibits autophagy by affecting calcium efflux. During the early stage of *M. tuberculosis* infection of macrophages, CtpF expression is elevated under conditions of macrophage stress, like hypoxia, elevated nitric oxide concentrations, and acidic environments. Elevated CtpF allows calcium efflux and activates mTOR, thus inhibiting autophagy and enhancing mycobacteria survival (Garg et al., 2020). Visfatin of host cells is associated with autophagy and ROS production during *M. tuberculosis* infection. Rv3242c of *M. tuberculosis*, encoding a phosphoribosyltransferase, can inhibit visfatin level in infected macrophages, thereby suppressing autophagy and ROS production. Rv3242c-expressing *M. smegmatis* activates MAPK and increases IL-10 production, suggesting that the inhibitory effect of Rv3242c on autophagy may be mediated through the MAPK pathway and IL-10 (Mohanty et al., 2015). KatG also inhibits host autophagy by reducing ROS. KatG, a catalase-peroxidase, is upregulated in the *M. tuberculosis* Beijing strain but not in H37Rv. The upregulated KatG neutralizes mitochondrial ROS generated during *M. tuberculosis* Beijing strain infection, thereby blocking autophagosome maturation and enhancing intracellular bacterial survival (Siregar et al., 2022). Protein kinase G (PKnG), the eukaryotic-like serine/threonine protein kinase, is secreted by pathogenic mycobacteria in infected macrophages, where it initiates autophagy but prevents fusion of the autophagosome and lysosome (Walburger et al., 2004; Ge et al., 2022). For autophagy initiation, PKnG binds directly to the pleckstrin homology domain of AKT, thereby reverting the inhibitory effect of AKT for autophagy. For autophagosome maturation inhibition, PKnG binds directly to host small GTPase RAB14 and inhibits the GTPase activity of RAB14. In addition, PKnG phosphorylates TBC1 domain family member 4 (TBC1D4), depriving it of the ability to activate RAB14 (Ge et al., 2022). Xenophagy against mycobacteria is associated with NADH dehydrogenase. NuoG, which encodes NADH dehydrogenase I subunit G, was previously recognized as an anti-apoptotic virulence factor of *M. tuberculosis*. However, knockout of NuoG allows more LC3 to be recruited to intracellular *M. bovis*, suggesting that NuoG is also involved in the inhibition of xenophagy (Gengenbacher et al., 2016). Regulation of NADH dehydrogenase determines the ability of different strains to induce bactericidal xenophagy.

Some other functional proteins apart from enzymes can also inhibit host autophagy. Latent mycobacteria infection is dependent on the balance between host and pathogenic bacteria, and heat shock proteins (HSPs) of *M. tuberculosis* play a key role in this process. Autophagosomes in HSP16.3 mutant *M. tuberculosis* infected macrophages are significantly more than that in wild strain infected cells, suggesting that HSP16.3 may increase bacterial survival by inhibiting host autophagy (Yang et al., 2018). Heparin-binding hemagglutinin (HBHA) of *M. tuberculosis* is another autophagy inhibitor. In A549 cells, HBHA avoids phagosome maturation by inhibiting LC3 expression, thereby increasing the survival of *M. smegmatis* expressing HBHA (Zheng et al., 2017). The inhibition of autophagy by *M. bovis* or Rv3416 of *M. tuberculosis* may be mediated through enhanced SUMOylation rather than ubiquitination. Inhibiting SUMOylation enhances the expression of autophagy markers and promotes autophagy, whereas *M. bovis* or Rv3416 of *M. tuberculosis* augment the SUMOylation of these autophagic molecules to suppress autophagy in infected bone marrow derived dendritic cells (BMDCs) (Anang et al., 2023). Mb3523c, a structural protein of *M. bovis* belonging to the Mce4 family, promotes bacterial evasion of clearance by inducing host CMA and ferroptosis. Mechanistically, Mb3523c protein promotes CMA by interacting with host HSP90 at Y237 and G241 sites, stabilizing LAMP2A on lysosomes to facilitate GPX4 degradation via the CMA pathway, thereby inducing ferroptosis to enhance bacterial pathogenicity and dissemination (Wang H. et al., 2025). Lipoprotein is a critical class of virulence proteins of *M. tuberculosis*, and its virulence is associated with the manipulation of autophagy. LprE mutant *M. tuberculosis* causes more autophagy-associated protein expression and more recruitment of lysosomal and phagosomal proteins in infected macrophages, suggesting that LprE inhibits autophagosome formation and fusion of autophagosomes with lysosomes. LprE inhibits phago-lysosome fusion because of downregulation of IL-12 and IL-22 (Padhi et al., 2019). As an important virulence protein of *M. tuberculosis*, the mechanism by which lipoproteins inhibit autophagy and increase intracellular bacterial load has been ambiguous and more investigation is needed.

Mycobacteria can also regulate autophagy by phosphorylating/dephosphorylating upstream molecules of autophagy. Phosphoproteome analysis revealed that *M. tuberculosis* infection induces extensive dephosphorylation of host proteins in macrophages, particularly in MAPK and PI3K signaling pathways critical for autophagy activation, whereas avirulent *M. bovis* infection elicits milder phosphorylation changes and preserves partial autophagic signaling (Choudhary et al., 2020). A recent study revealed the role of potassium ion in the induction of autophagy. Mycobacterial infection can upregulate the surface expression of potassium ion channel Kir2.1 in epithelial cells and macrophages. Inhibition of Kir2.1 can promote autophagy and apoptosis by enhancing oxidative burst and activating the MAPK/NF-κB pathway, significantly reducing bacterial survival, suggesting that Kir2.1 may assist mycobacterial immune escape by regulating ion homeostasis (Sinha et al., 2024). However, the specific factors by which mycobacteria regulate host protein phosphorylation status and enhance potassium channel protein expression remain to be elucidated.

Although many studies have shown that autophagy is an important part of the host’s response to clear mycobacteria, a growing amount of research has challenged this opinion. On the one hand, in addition to autophagy, autophagy genes are also involved in non-autophagic processes (Galluzzi and Green, 2019), and it is difficult to conclude that the changes in intracellular mycobacteria survival after the deletion of a certain host autophagy gene are necessarily related to autophagy. For instance, an ingenious investigation showed no change in mycobacteria proliferation after abrogating host autophagy by knocking out the autophagy genes ATG3, ATG7, ATG12, ATG14, or ATG16l1, suggesting that autophagy is dispensable for inhibiting mycobacteria proliferation. Concurrently, the deletion of ATG5 resulted in a marked increase in bacterial proliferation, ultimately leading to the demise of all infected mice. The research team put forth the hypothesis that ATG5 exerts its antimycobacterial effects through preventing neutrophil-mediated immunopathology, rather than autophagy (Kimmey et al., 2015). So, further investigation is required to elucidate the precise function of autophagy genes in combating mycobacteria. On the other hand, certain virulence factors associated with mycobacteria species have been observed to induce autophagosome formation while simultaneously inhibiting the fusion of these autophagosomes with lysosomes. In addition to the previously mentioned PKnG, ESX-1 secretory system can also be considered as a virulent factor of this kind. ESX-1 secretory system is necessary for the induction of host xenophagy during early stages of *M. tuberculosis* infection (Watson et al., 2012, 2015); however, it also inhibits the fusion of autophagosomes with lysosomes during the late infection stages (Romagnoli et al., 2012; Chandra et al., 2015). The dysregulation of organelles has the potential to induce the secretion of antimicrobial and inflammatory cytokines (Zhou et al., 2011; Liao et al., 2019) and autophagy may assist in the removal of dysregulated organelles, thereby reducing the secretion of these cytokines (Nakahira et al., 2011; Di Rita et al., 2021; Han et al., 2021).In the event of *M. tuberculosis* inhibiting the entirety of the autophagic flux, this would result in dysregulated organelles remaining incompletely cleared and an increase in cytokine secretion. To prevent the maturation of bacteriophage-containing autophagosomes, while allowing for the maturation of bacteriophage-free autophagosomes, *M. tuberculosis* employed a subtle strategy to expel Rab7 from *M. tuberculosis*-containing autophagosomes. This approach ensured the uninterrupted progression of entire autophagic fluxes (Chandra and Kumar, 2016). This evidence suggests that autophagosomes may serve as a niche for mycobacteria replication.

## 5 Agents targeting autophagy against mycobacteria

The ability of mycobacteria to inhibit autophagy may be negatively correlated with their pathogenicity (Gonzalez-Orozco et al., 2022). Faced with the sophisticated strategies employed by mycobacteria to regulate autophagy for survival, the development of therapeutic interventions that modulate autophagy in the host has emerged as a promising approach in combating mycobacterial infections (**Table 2**). This type of approach is generally called HDT.

**Table 2:** Agents targeting autophagy against mycobacteria.

Agents	Mycobacteria	Study setting	Mechanism	Reference
2062	*M. tuberculosis*	*In vivo*; *In vitro*	Improves autophagy by TFEB	(Bryk et al., 2020)
Ambroxol	*M. tuberculosis*	*In vivo*; *In vitro*	Increases autophagosomes production	(Choi et al., 2018)
Amoxapine	*M. bovis*; *M. tuberculosis*	*In vivo*; *In vitro*	Inhibits mTOR	(Wang et al., 2022)
Baicalin	*M. tuberculosis*	*In vitro*	Inhibits PI3K/AKT/mTOR pathway	(Zhang et al., 2017)
Bazedoxifene	*M. tuberculosis*	*In vitro*	Increases ROS and phosphorylation of AKT/mTOR signaling	(Ouyang et al., 2020)
Bedaquiline	*M. tuberculosis*	*In vitro*	Increases ROS	(Genestet et al., 2018; Giraud-Gatineau et al., 2020)
Carbamazepine	*M. tuberculosis*	*In vivo*; *In vitro*	Activates AMPK and induces autophagy in an mTOR independent manner	(Cárdenas-Rodríguez et al., 2013; Schiebler et al., 2015)
Degarelix	*M. marinum*; *M. tuberculosis*	*In vivo*; *In vitro*	Increases IFN-γ expression and autophagy initiation	(Li et al., 2024)
Everolimus	*M. tuberculosis*	*In vitro*	Inhibits mTOR	(Ashley et al., 2020)
Furamidine	*M. bovis*; *M. smegmatis*	*In vitro*	Activates Ca^2+^/AMPK/SIRT1/FOXO3a pathway	(Patel et al., 2025)
Gliotoxin	*M. tuberculosis*	*In vitro*	Increases Atg5 expression and autophagy initiation	(Fu et al., 2023)
GP	*M. tuberculosis*	*In vitro*	Enhances ROS and NO production and autophagolysosome maturation	(Upadhyay et al., 2019)
H_2_S	*M. tuberculosis*	*In vitro*	Activates CCAR2-SIRT1-LC3 axis	(Iqbal et al., 2021)
Ibrutinib	*M. tuberculosis*	*In vivo*; *In vitro*	Facilitates phagosome-lysosome fusion	(Hu et al., 2020)
Imiquimod	*M. tuberculosis*	*In vitro*	Increases ROS and NO production	(Lee et al., 2020)
Isoniazid	*M. tuberculosis*; *M. marinum*	*In vivo*; *In vitro*	Increases ROS; Activates Ca^2+^-AMPK pathway	(Kim et al., 2012)
Linezolid	*M. tuberculosis*	*In vitro*	Reduce the inhibition of autophagy by bacteria	(Genestet et al., 2018)
Loperamide	*M. tuberculosis*	*In vitro*	Increases colocalization of LC3 with *M. tuberculosis*	(Juárez et al., 2016)
Metformin	*M. tuberculosis*	*In vitro*	Increases AMPK expression, inducing phosphorylation of ULK1	(Singhal et al., 2014)
Nitazoxanide	*M. tuberculosis*	*In vivo*; *In vitro*	Inhibits mTOR	(Lam et al., 2012)
Pyrazinamide	*M. tuberculosis*; *M. marinum*	*In vivo*; *In vitro*	Increases ROS; Activates Ca^2+^-AMPK pathway	(Kim et al., 2012)
Rapamycin	*M. avium*, *M. smegmatis*, *M. tuberculosis*	*In vivo*; *In vitro*	Inhibits mTOR	(Greenstein et al., 2008; Bhatt et al., 2021)
Resveratrol	*M. tuberculosis*	*In vivo*; *In vitro*	Inhibits mTOR	(Liu et al., 2010; Park et al., 2016; Cheng et al., 2017)
Rosuvastatin	*M. tuberculosis*	*In vivo*; *In vitro*	Reduces membrane cholesterol levels, increasing autophagic flux	(Parihar et al., 2014)
Rufomycin	M. abscessus	*In vivo*; *In vitro*	Improves autophagy by TFEB	(Park et al., 2021)
Se NPs	*M. tuberculosis*; *M. bovis*	*In vitro*	Inhibits mTOR; Increases ROS	(Pi et al., 2020)
Simvastatin	*M. tuberculosis*	*In vivo*; *In vitro*	Reduces membrane cholesterol levels, increasing autophagic flux	(Parihar et al., 2014)
Soybean lectin	*M. bovis*	*In vitro*	Activates JAK2/STAT3/Mcl-1 and P2RX7-NF-κB pathway	(Mishra et al., 2021, 2023)
Tamoxifen	*M. marinum*; *M. tuberculosis*	*In vivo*; *In vitro*	Improves autophagy by TFEB	(Boland et al., 2023)
Trehalose	*M. avium*; *M. fortuitum*	*In vivo*; *In vitro*	Activates PIKFYVE-MCOLN1-TFEB pathway	(Sharma V. et al., 2021)
Valproic acid	*M. tuberculosis*	*In vitro*	Increases colocalization of LC3 with *M. tuberculosis*	(Schiebler et al., 2015)
Vitamin A	*M. tuberculosis*	*In vitro*	Metabolite of vitamin A increases autophagy through the STING/TBK1/IRF3 axis	(Coleman et al., 2018)
Vitamin D	*M. tuberculosis*	*In vitro*	Activates autophagy through PI3K, calcium, cathelicidin, calmodulin-dependent kinase kinase-beta and AMP-activated protein kinase	(Jo, 2010)
ZnO NPs	*M. tuberculosis*; *M. bovis*	*In vivo*; *In vitro*	Increases LC3-II/I ratio and the decreases the SQSTM1/p62 level	(Geng et al., 2023)
ZnO-Se NPs	*M. tuberculosis*	*In vitro*	Increases intracellular ROS; Disrupts mitochondrial membrane potential; Inhibits the PI3K/AKT/mTOR signaling	(Lin et al., 2022)

### 5.1 Classic autophagy inducers

Rapamycin is one of the most extensively researched autophagy inducers. Indeed, in an experimental context, rapamycin demonstrated notable inhibitory activity against mycobacterial infections (Greenstein et al., 2008; Bhatt et al., 2021). However, the absorption of rapamycin in the gastrointestinal tract exhibits considerable fluctuations, necessitating monitoring during administration and causing significant inconvenience in the clinical use of the drug. The administration of rapamycin has been observed to induce interstitial pneumonitis, which may potentially offset some of the positive antimicrobial effects observed in cases of mycobacteria pulmonale infection. Additionally, rifampicin, a standard treatment for tuberculosis, stimulates expression of the hepatic enzyme CYP3A4, which metabolizes rapamycin. These factors restrict the clinical application of rapamycin in the treatment of mycobacteria infections. Everolimus, as a rapamycin analog, also targets mTOR and can significantly reduce the intracellular *M. tuberculosis* burden in granulomas (Ashley et al., 2020). However, experiments in *M. tuberculosis*-infected THP-1 macrophages, a different picture emerged. Everolimus stimulated autophagy by increasing ROS and autophagosome formation, but it did not promote autolysosome generation or significantly inhibit intracellular *M. tuberculosis* replication (Bianco et al., 2023). This contradiction highlights the importance of more preclinical experiments when everolimus comes to drug repurpose. Ibrutinib, as another agent targeting the mTOR pathway, can significantly increase the colocalization of LC3 and *M. tuberculosis* as well as auto-lysosome fusion, significantly reducing the *M. tuberculosis* load in the mediastinal node and spleen of infected mice (Hu et al., 2020). If further research can confirm that everolimus and ibrutinib do not have the clinical limitations of rapamycin, then these agents could be promising HDT drugs.

Metformin, renowned for its AMPK-mediated inhibitive role in mTOR signaling pathway, is prevalent in managing type-2 diabetes, and it triggers ROS generation, phagosome maturation, autophagy *in vitro* (Singhal et al., 2014; Yang, 2017; Russell et al., 2019). The administration of metformin to healthy human volunteers led to a notable downregulation of genes associated with the mTOR signaling pathway and an upregulation of genes associated with phagocytosis and the generation of ROS. *In vitro*, the metformin treatment in mononuclear cells isolated from peripheral blood of healthy donors resulted in enhancement of cellular metabolism and suppression of mTOR downstream effectors, p70S6K and 4EBP1 (Lachmandas et al., 2019). Several clinical studies have shown that the use of metformin effectively reduces the risk of active tuberculosis and mortality in patients with diabetes mellitus and effectively promotes sputum culture conversion in cavitary pulmonary patients (Degner et al., 2018; Lee M. et al., 2018; Lee Y. et al., 2018). Consistent with the above studies, Singhal et al. showed that metformin restricted the growth of drug-resistant *M. tuberculosis* strains in an AMPK-dependent manner, alleviated lung lesions in infected mice, increased ROS production and phagosome maturation, and improved the efficacy of anti-tuberculosis drugs (Singhal et al., 2014). However, another experiment conducted by Dutta et al. showed that the combination of metformin did not improve the activity of first-line anti-tuberculosis drugs in mice (Dutta et al., 2017). This discrepancy cannot be explained by the dose of metformin because the same dose was used in both experiments (250 mg/kg). It is speculated that this difference may be related to the different experimental mouse strains used in the two experiments. Given that Dutta et al. used RHZE and rifampicin while Singhal et al. used isoniazid or ethambutol, another seemingly more plausible theory is that the powerful drug RHZE utilized in the Dutta’s experiment masked the effects of metformin, or that rifampicin sped up the clearance of metformin.

As classic autophagy inducers, rapamycin and metformin have shown the potential to eliminate mycobacteria, but issues such as side effects, drug interactions and consistency of therapeutic efficacy have restricted their clinical application. Future research needs to focus on analogue optimization, delivery system innovation and combined therapeutic strategies to promote the clinical transformation of host-directed therapy in the fight against mycobacterial infections.

### 5.2 Antibiotics

Commonly used anti-tuberculosis antibiotics isoniazid and pyrazinamide have the effect of inducing autophagy and ROS production in host cells infected by *M. tuberculosis* (Kim et al., 2012). Isoniazid and pyrazinamide increase cellular and mitochondrial ROS and facilitate phagosome-lysosome fusion in *M. tuberculosis*-infected host cells. *In vivo*, its antimycobacterial efficacy relies on host autophagy, as autophagy-defective models show reduced survival in the context of administering antibiotics (Kim et al., 2012). Another antibiotic that can induce host cell autophagy is nitazoxanide. Nitazoxanide can directly restrain *M. tuberculosis* proliferation *in vitro*, and it exerts a stronger inhibitory effect within host cells. Mechanistically, nitazoxanide suppresses the quinone oxidoreductase in host cells, thereby blocking the mTOR signaling pathway and enhancing autophagy (Lam et al., 2012). Bedaquiline and 2062, respectively a new antibiotic and a small molecule substance, can both increase autophagy and phagosome-lysosome fusion by activating TFEB (Bryk et al., 2020; Giraud-Gatineau et al., 2020). Given the effectiveness of autophagy in clearing intracellular bacteria, it is worth exploring whether first-line anti-tuberculosis drugs, which have abundant safety evaluation data, can be used as adjunctive medication against NTMs, even if they do not have significant activity direct against NTMs.

Rufomycin can partially restore the expression and nuclear translocation of TFEB in BMDMs infected with the *M. abscessus*, activate the mRNA expression of autophagy/lysosome-related genes downstream of TFEB, and increase the colocalization of *M. abscessus* phagosomes and lysosomes, indicating that rufomycin can enhance autophagy during *M. abscessus* infection (Park et al., 2021).

Antibiotic-induced autophagy is not necessarily due to direct stimulation of host cells. After macrophages are infected with *M. tuberculosis* pre-treated with rifampicin, linezolid or bedaquiline, autophagy activation and efficacy are enhanced, indicating that antibiotics can promote host cell autophagic clearance by altering bacterial protein synthesis or energy metabolism (Genestet et al., 2018). Exposure to isoniazid, bedaquiline, rifampicin, and O-floxacin causes *M. tuberculosis* to exhibit higher NADH: NAD^+^ ratios in infected macrophages, facilitating the production of more ROS (Bhat et al., 2016). ROS is an inducer of autophagy. The ROS produced by *M. tuberculosis* exposed to antibiotics may be released into the infected host cells, thereby inducing autophagy. It is speculated that this is at least part of the mechanism by which antibiotics are effective against *M. tuberculosis* (Piccaro et al., 2014).

Although some commonly used anti-tuberculosis antibiotics show the potential to induce autophagy, the association between their antibacterial activity and autophagy remains unclear, and the mechanism of action needs to be refined. In the future, efforts should be focused on mechanism analysis and exploration of the potential for adjuvant therapy.

### 5.3 Other chemical agents

Multidrug-resistant *M. tuberculosis* has evolved mechanisms to resist autophagy, resulting in basal autophagy being insufficient to eliminate the bacteria. The anticonvulsant drug carbamazepine can induce sufficient autophagy to clear *M. tuberculosis* through a pathway that does not rely on mTOR but reduces intracellular myoinositol (Schiebler et al., 2015). In addition, carbamazepine can also activate AMPK. Although this result does not come from *M. tuberculosis* infection experiments, it does not rule out the possibility that AMPK is involved in the process of carbamazepine-induced autophagy in infected cells (Cárdenas-Rodríguez et al., 2013). Another anticonvulsant medication valproic acid and the anti-diarrhea drug loperamide can also enhance autophagy, and this enhancement is evidenced by the increased co-localization of LC3 and *M. tuberculosis* (Schiebler et al., 2015; Juárez et al., 2016). Furamidine, a minor groove binder of DNA, is also an autophagy inducer that acts on AMPK. Furamidine induces autophagy in differentiated THP-1 cells via the Ca^2+^/AMPK/silent mating type information regulation 1 (SIRT1)/forkhead box O3 (FOXO3a) signaling pathway, reducing intracellular *M. tuberculosis* burden through enhanced autophagic flux, as evidenced by LC3-II conversion, autophagic vacuole accumulation, and activation of autophagic markers (Patel et al., 2025).

Degarelix, a synthetic decapeptide GnRH antagonist, inhibits luteinizing and follicle-stimulating hormone production to reduce testosterone and estrogen synthesis, and was clinically approved for prostate cancer treatment (Devos et al., 2023). A recent study shows that degarelix induces autophagy initiation in macrophages via a PI3K-dependent pathway, potentially synergizing with IFN-γ upregulation to enhance antimycobacterial activity, without altering classical autophagic flux. These findings highlight degarelix as a novel host-directed therapeutic candidate for TB by targeting early autophagic mechanisms (Li et al., 2024). Gliotoxin, a metabolite derived from marine fungi, is a bioactive compound with potential antibacterial properties. Experiments showed that gliotoxin significantly increased the LC3-II/LC3-I ratio and ATG5 expression to promote autophagy, and the autophagy inhibitor 3-MA could suppress the induced autophagy and restore gliotoxin-inhibited *M. tuberculosis* infection. Since 3-MA mainly inhibits the initiation of autophagy, gliotoxin might suppress *M. tuberculosis* infection in macrophages by promoting autophagy initiation (Fu et al., 2023). The study found that amoxapine can inhibit mTOR activation, induce autophagy in macrophages, increase the level of LC3B-II, promote autophagosome formation without affecting autophagic flux. After inhibiting autophagy with 3-MA or knocking down ATG16L1, the antibacterial effect of amoxapine against intracellular mycobacteria was significantly weakened, indicating that autophagy plays a crucial role in the process of amoxapine inhibiting the growth of intracellular mycobacteria (Wang et al., 2022). Dimethyl itaconate is another agent that can induce autophagy initiation, but it has not been approved for clinical treatment. Dimethyl itaconate can enhance autophagic flux and phagolysosomal fusion in macrophages infected with mycobacteria, as evidenced by increased autophagic LC3-II accumulation and bacterial colocalization with lysosomes (Kim et al., 2023).

The decrease of membrane cholesterol levels mediated by simvastatin and rosuvastatin promotes autophagy and phagosomal maturation, reduces bacterial load and lung burdens, and improves histopathologic changes (Parihar et al., 2014). Fluvastatin, another member of statins, possesses moderate antimycobacterial activity against *M. tuberculosis* (Battah et al., 2019). It is plausible to hypothesize that fluvastatin reduces membrane cholesterol synthesis by inhibiting hydroxy-methyl-glutaryl-CoA (HMG-CoA) reductase, thereby promoting phagosomal maturation and autophagy to reduce intracellular bacterial burdens in infected host cells.

Selective estrogen receptor modulators, such as tamoxifen and bazedoxifene, have been shown to exhibit the effect of inhibiting the growth of *M. tuberculosis* within macrophages. *In vitro* and *in vivo*, tamoxifen increases autophagy related vesicles, enhances mycobacterial localization in lysosomes, and its antimycobacterial effect is associated with autophagy -lysosomal pathway modulation, as inhibition of lysosomal activity reduces its efficacy (Boland et al., 2023). Treatment with bazedoxifene increases autophagosome formation and the expression of LC3B-II protein in infected macrophages. This indicates that the anti-mycobacterial activity of bazedoxifene might be related to autophagy. Autophagy indeced by bazedoxifene is associated with an increase in ROS and the phosphorylation of the AKT/mTOR signaling pathway (Ouyang et al., 2020).

Imiquimod, a drug for treating superficial basal cell carcinoma, induces BNIP3-mediated mitophagy through stimulating TLR7 of macrophage. On the other hand, imiquimod induces NO production through the GSK-3β-mediated signaling pathway, which leads to autophagy in the late stage. The autophagy triggered by imiquimod can effectively eliminate *M. tuberculosis* within macrophages (Lee et al., 2020).

Ambroxol, a lead compound identified from screens for autophagy-inducing drugs, represents a potential host-directed therapy adjunct to conventional antibiotic chemotherapy against *M. tuberculosis*. At clinically relevant doses, ambroxol elicited autophagy both *in vitro* and *in vivo*, thereby promoting the elimination of mycobacteria by host. Moreover, ambroxol additionally potentiated the antimicrobial activity of rifampin *in vivo*, demonstrating synergistic effects in combating mycobacterial infection (Choi et al., 2018).

A study found that H_2_S can sulfhydrate GAPDH and cause its translocation to the nucleus, where it interacts with cell cycle and apoptosis regulator 2 (CCAR2), disrupts the CCAR2-SIRT1 complex, activating SIRT1. Subsequently, SIRT1 deacetylates LC3B, enabling its translocation to the cytoplasm and inducing autophagy. Moreover, H_2_S-induced autophagy can promote the trafficking of *M. tuberculosis* to lysosomes and restrict its intracellular growth. This process depends on the sulfhydration of GAPDH, and SIRT1 is crucial for H_2_S-induced autophagy and the inhibition of *M. tuberculosis* growth (Iqbal et al., 2021). Since GAPDH is widely present in various types of cells, autophagy dependent on the sulfhydration of GAPDH may occur in various cells, enabling H_2_S to serve as a broad-spectrum autophagy inducer. However, considering the toxicity of H_2_S, developing low-toxicity analogs might be a better strategy.

Clinically approved chemical drugs with validated safety profiles, particularly those indicated for patients with comorbidities involving their primary approved indications and mycobacterial infections, warrant mechanistic exploration of their synergistic therapeutic potential and precise druggable targets to advance rational combination therapies.

### 5.4 Natural products

The catalytic product of vitamin D-1-hydroxylase, calcitriol, exhibits antibacterial effects. By activating TLR to upregulate vitamin D-1-hydroxylase, cathelicidin can be induced, indicating that calcitriol may exert its antibacterial effect through cathelicidin (Liu et al., 2006; Periyasamy et al., 2020). Cathelicidin may induce autophagy through pathways such as TFEB, AMPK, ULK1 and MAPK (Ikutama et al., 2023; Xi et al., 2024; Yang et al., 2024).

Although studies have shown that vitamin D can induce autophagy through multiple pathways (Jo, 2010), based on meta-analyses from multiple randomized controlled trials, vitamin D does not have consistent efficacy in the HDT against tuberculosis (Wu et al., 2018; Jolliffe et al., 2019; Tang et al., 2020). Although a meta-analysis shows that vitamin D deficiency may be a risk factor for tuberculosis (Huang et al., 2017), high-dose use of vitamin D cannot effectively accelerate the sputum culture conversion process in the entire trial population, but it is only effective in MDR-TB cases or patients with a specific genotype, such as polymorphisms in the vitamin D receptor gene (Tukvadze et al., 2015; Ganmaa et al., 2017). The combined use of vitamin D and phenylbutyrate (PBA) can induce the expression of cathelicidin and cathelicidin-induced autophagy in macrophages, reducing *M. tuberculosis* proliferation (Coussens et al., 2015; Rekha et al., 2015). However, the effect of this combined therapy on increasing cathelicidin expression is only observed at very specific doses of PBA (Mily et al., 2013). It is possible that due to the specific doses of PBA, multiple randomized controlled trials of the combined use of vitamin D and PBA cannot yield consistent results (Mily et al., 2015; Bekele et al., 2018), leading to vitamin D not being able to become an effective HDT for treating mycobacterial infections.

Similar to vitamin D, vitamin A may be involved in host resistance to *M. tuberculosis*. Vitamin A deficiency was significantly associated with elevated tuberculosis incidence among HIV-infected individuals (Tenforde et al., 2017). Mechanistically, the metabolite of vitamin A, all-trans retinoic acid, increases autophagy through the STING/TBK1/IRF3 axis, enhancing the colocalization of *M. tuberculosis* autophagic vesicles and acidified lysosomes (Coleman et al., 2018). Although vitamin A can reduce the mycobacterial load in mice, there is a shortage of consistent evidence regarding its benefits in tuberculosis patients (Karyadi et al., 2002; Lawson et al., 2010; Visser et al., 2011; Wang J. et al., 2020). As a result, whether vitamin A can be used as an adjunct therapy for tuberculosis remains indeterminate.

Many studies have confirmed the protective effects of autophagy in alleviating the excessive inflammatory response caused by *M. tuberculosis* (Castillo et al., 2012). Excessive activation of inflammasomes can lead to the excessive secretion of pro-inflammatory cytokines. The excessive secretion of these cytokines triggered by *M. tuberculosis* infection can lead to lung damage, which is detrimental to recovery. Excessive inflammasome activation can be mitigated by the autophagic clearance of endogenous stimuli and inflammasome components (Nakahira et al., 2011; Zhou et al., 2011; Shi et al., 2012). In this way, autophagy exerts protective effects in mycobacterial infections from two aspects: on one hand, autophagy can engulf and eliminate pathogens; on the other hand, autophagy can reduce inflammatory damage caused by the infection. Baicalin induces autophagy in *M. tuberculosis*-infected macrophages through the PI3K/AKT/mTOR pathway while simultaneously reducing inflammasome activation by inhibiting the PI3K/AKT/NF-κB pathway. Induced autophagy can also clear inflammasomes, thereby reducing the production of inflammatory cytokines. Therefore, baicalin can be considered a candidate drug for eliminating *M. tuberculosis* and reducing inflammatory lung damage (Zhang et al., 2017).

Resveratrol induces autophagy by directly binding to the ATP-binding pocket of mTOR or promoting the interaction between mTOR and its inhibitor DEPTOR, thereby suppressing mTOR signaling (Liu et al., 2010; Park et al., 2016). Resveratrol, a SIRT1 activator, reduces intracellular growth of drug-susceptible and drug-resistant *M. tuberculosis* strains by inducing phagosome-lysosome fusion and autophagy in a SIRT1-dependent manner while dampening *M. tuberculosis*-mediated inflammatory responses via deacetylation of RelA/p65. In *M. tuberculosis*-infected mice, Resveratrol ameliorates lung pathology, reduces chronic inflammation, and enhances the efficacy of anti-TB drugs, highlighting its potential as a host-directed therapy for tuberculosis (Cheng et al., 2017).

Granulocyte-macrophage colony-stimulating factor (GM-CSF) is an agent that can reduce the *M. tuberculosis* and Mav burden. In hosts infected with either *M. tuberculosis* or Mav, GM-CSF enhanced phagosome maturation and inhibited bacterial growth. Autophagy might play a significant role in GM-CSF activated immunity against *M. tuberculosis* and NTM (Kedzierska et al., 2000; Nannini et al., 2002; de Silva et al., 2007; Hariadi and Blackwood, 2017).

Soybean lectin, a glycoprotein isolated from soybean seeds with immunomodulatory activity, triggers IL-6 secretion through the P2RX7-dependent PI3K/AKT/CREB pathway, which activates the JAK2/STAT3/Mcl-1 pathway in an autocrine manner to induce autophagy and eliminate intracellular mycobacteria in differentiated THP-1 macrophages (Mishra et al., 2023). Furthermore, there is also a study showing that soybean lectin induces autophagy in differentiated THP-1 cells via a P2RX7-NF-κB-dependent pathway, increasing ROS generation and enhancing autophagic flux, thereby restricting intracellular *M. tuberculosis* growth in infected cells (Mishra et al., 2021).

Trehalose is a naturally occurring disaccharide with mTOR-independent autophagy-inducing properties. HIV infection was shown to inhibit autophagy flux in macrophages, promoting the survival of *M. tuberculosis* and NTM. Conversely, trehalose induced autophagy via a phosphoinositide kinase-FYVE finger containing (PIKFYVE)- Transient receptor potential channel mucolipin-1 (TRPML1)-dependent pathway, promoting TFEB nuclear translocation, upregulating autophagy and lysosomal genes, enhancing phagosome-lysosome colocalization, and thus restricting intracellular mycobacterial survival during both single and HIV co-infection (Sharma V. et al., 2021).

Exploring efficacy variability from the perspective of genetic polymorphisms and developing genotype-matched optimized protocols may facilitate the promotion of natural products in mycobacterial disease treatment.

### 5.5 Nanoparticles

ZnO nanoparticles (ZnO NPs) exhibit antibacterial effects against various *M. tuberculosis* strains, including multidrug-resistant strains. Moreover, ZnO NPs can dose-dependently induce autophagy and reduce mycobacteria load within macrophages. However, high-dose ZnO NPs can cause ferroptosis. Studies have shown that the combination of ferroptosis inhibitor and ZnO NPs can induce sufficient levels of autophagy to eliminate *M. tuberculosis* while avoiding acute lung injury that may be caused by ferroptosis *in vivo* (Geng et al., 2023).

Se nanoparticles (Se NPs) are another type of nanoparticles that not only have antibacterial activity (Huang et al., 2019; Estevez et al., 2020) but also activate host cell immune responses, such as autophagy (Pi et al., 2020). The autophagy activated by Se NPs is related to the alteration of ROS production, mitochondrial membrane potential, and the PI3K/AKT/mTOR signaling pathway, playing a crucial role in clearing intracellular *M. tuberculosis*.

The novel zinc oxide selenium nanoparticles (ZnO-Se NPs) made by combining ZnO NPs and Se NPs, like ZnO NPs or Se NPs, have the ability to directly suppress extracellular *M. tuberculosis* and stimulate the host cell immune response for intracellular *M. tuberculosis* elimination. ZnO-Se NPs induce host cell autophagy by increasing intracellular ROS, disturbing mitochondrial membrane potential, and blocking the PI3K/AKT/mTOR signaling pathway (Lin et al., 2022). Thus, nanoparticles like ZnO NPs, Se NPs and ZnO-Se NPs, which combine direct bactericidal and autophagy-activating effects, have the potential to develop into new therapeutic agents against mycobacteria.

Similar to ZnO-Se NPs, β-Glucan particles (GP) can trigger strong immune responses, including autophagy, within *M. tuberculosis*-infected macrophages. Unlike ZnO-Se NPs, GP cannot directly eliminate bacteria, but it can serve as a delivery system to transport Rifabutin (RB) nanoparticles into macrophages, enhancing the bactericidal efficacy of RB (Upadhyay et al., 2019).

Graphene oxide (GO) nanoparticles cannot directly kill bacteria extracellularly, nor can they induce autophagy or other immune responses within *M. tuberculosis*-infected macrophages. However, they can serve as drug delivery vehicles to transport drugs with autophagy-inducing or bactericidal activities into host cells, thereby achieving and maintaining high intracellular drug concentrations, which facilitates the clearance of *M. tuberculosis* (Saifullah et al., 2017; Pi et al., 2019; De Maio et al., 2020). Curcumin is an effective autophagy inducer, but poor bioavailability limits its application prospects (Lopresti, 2018). Encapsulating curcumin in a polylactic acid-glycolic acid shell to obtain polymerized *in situ* curcumin nanoparticles can effectively improve the bioavailability of curcumin and significantly increase autophagy in *M. tuberculosis*-infected macrophages (Gupta et al., 2023).

In addition to chemical drugs, nanoparticles can also deliver nucleic acids to induce autophagy. Nucleic acids are widely involved in the regulation of autophagy in host cells infected by *M. tuberculosis*. Liposomal nanoparticles can simultaneously deliver chemical drugs and nucleic acids into cells, combining the advantages of chemotherapy, nanotechnology, and nucleic acid therapy for autophagy induction. Delivering a certain concentration of siRNA and anti-tuberculosis drugs into THP-1 cells infected with *M. tuberculosis* through liposomal nanoparticles can significantly increase the proportion of autophagic cells (Niu et al., 2015).

Nanomedicines demonstrate remarkable advantages in therapeutic, preventive, and diagnostic applications for diseases. Numerous nanomaterials exhibit promising potential in enhancing mycobacterial clearance through direct or indirect modulation of autophagy. Continued exploration of their anti-tuberculosis mechanisms, *in vivo* metabolic profiles, biosafety, and degradation patterns will facilitate the development of novel therapeutic strategies against mycobacteria.

## 6 Conclusion and prospects

Autophagy, a conserved cellular mechanism, plays a pivotal role in the host’s defense against mycobacterial infections, encompassing both non-selective and selective pathways. However, mycobacteria, including *M. tuberculosis* and NTM, have evolved sophisticated strategies to subvert autophagy, such as deploying PE/PPE family proteins, SapM, and ESX-1 secretion systems to inhibit autophagosome-lysosome fusion or activate mTOR signaling.

HDT targeting autophagy has emerged as a promising approach. Classic autophagy inducers, antibiotics, natural products and nanomaterials have shown efficacy in preclinical models by enhancing autophagic flux or bacterial clearance. Notably, nanomedicines offer dual advantages of direct bactericidal activity and autophagy induction, overcoming limitations like poor bioavailability.

Key challenges and future directions include:

Mechanistic clarification. Disentangling the specific roles of autophagy genes from their non-autophagic functions and understanding how mycobacterial virulence factors differentially modulate autophagy at distinct infection stages.

Multi-omics integrated modeling. Integrating transcriptome, proteome, and metabolome data to construct a predictive biomarker model for autophagy regulation therapy.

Therapeutic optimization. Developing autophagy-inducing agents with minimal side effects and addressing drug interactions. Combination therapies and nanocarrier-based delivery systems may enhance efficacy and reduce toxicity.

Translational research. Advancing preclinical findings to clinical trials, particularly for NTM and drug-resistant tuberculosis. Genotype-specific therapies and personalized medicine approaches warrant exploration.

In summary, autophagy represents a critical nexus of host-pathogen interaction in mycobacterial diseases. Future research integrating molecular mechanisms, innovative drug delivery, and clinical translation will unlock its full potential for developing effective, host-centric therapies against these persistent pathogens.
